# Polymorphism of BaTeO_3_ under High Pressure: Single-Crystal Structure Analysis and Characterization of HP-BaTeO_3_

**DOI:** 10.1021/acs.cgd.5c01378

**Published:** 2026-01-22

**Authors:** Benjamin J. Pullicino, Stefan Schwarzmüller, Gunter Heymann

**Affiliations:** Institut für Allgemeine, Anorganische und Theoretische Chemie, 27255 Universität Innsbruck, Innrain 80−82, Innsbruck A-6020, Austria

## Abstract

A new high-pressure polymorph of barium tellurate, HP-BaTeO_3_, was synthesized using multianvil high-pressure/high-temperature techniques (4 GPa, 900 °C). The compound crystallizes in the monoclinic space group *P*2_1_/*c* and consists of stacked trigonal pyramidal [TeO_3_]^2–^ units interconnected by secondary bonds. Structural analysis identifies significant differences between HP-BaTeO_3_ and its ambient-pressure polymorph, BaTeO_3_(I), including a doubling of the *c*-axis and additional secondary bonding within the *bc* plane. The optical properties of HP-BaTeO_3_ were investigated using ultraviolet–visible spectroscopy, revealing a widened bandgap compared to BaTeO_3_(I), attributed to changes in orbital overlap and lone pair orientation. Thermal analysis and high-temperature powder X-ray diffraction confirmed the metastable nature of HP-BaTeO_3_, with a phase transition to BaTeO_3_(I) occurring at approximately 550 °C. This study highlights the structural and electronic modifications induced by high-pressure synthesis and provides insights into the relationship between the two polymorphs.

## Graphical abstract

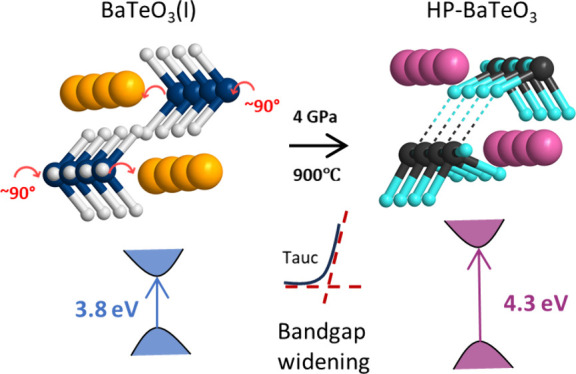

## 1 Introduction

The search for noncentrosymmetric crystal structures has spurred on the discovery of new oxotellurate phases. This is particularly due to the potential of the acentric [Te^4+^O_*n*_] polyhedra in forming such polar configurations,^1–4^ which are essential for the development of new nonlinear optics, pyroelectrics, and piezoelectrics.^5^ The coordinative flexibility of Te^4+^ with oxygen and multiple Te oxidation states contribute profoundly to the rich crystal chemistry of oxotellurates, along with the corner-sharing and edge-sharing ability of tellurate polyhedra.^6^ Application of high pressure combined with high temperature via multianvil techniques frequently produces new metastable phases. Recently, high-pressure techniques have been employed for the synthesis of new oxotellurates as a novel route toward noncentrosymmetric crystal structures^7–9^ and for a better understanding of the crystal chemistry of the oxotellurates.^10–12^ In the aim of carrying out explorative syntheses of alkaline-earth tellurates, this study reveals the formation of a new, high-pressure phase of BaTeO_3_ (HP-BaTeO_3_) through high-pressure/high-temperature (HP/HT) techniques.

Several barium tellurate­(IV) phases are currently known, and most of them have been synthesized using conventional high-temperature techniques. These include BaTe_2_O_5_ in monoclinic *P*2_1_/*c*,^13^ orthorhombic BaTe_3_O_8_ (unknown S.G.),^13^ tetragonal Ba_2_TeO_4_ in *P*4_1_2_1_2_1_,^14^ Ba_3_Te_4_O_11_ ^15^ in triclinic *P*, and two modifications of BaTeO_3_: BaTeO_3_(I)^16^ in monoclinic *P*2_1_/*m* and BaTeO_3_(II)^17^ in orthorhombic *Pnma*. Only X-ray powder data are known for the first three barium tellurates mentioned. Furthermore, BaTe_2_O_5_ and Ba_2_Te_3_O_8_ do not seem to be isotypic to their alkaline-earth relatives with the same sum formula. Different unit-cell parameters have been recorded for these phases, and Ba_2_TeO_4_ appears to have a unique stoichiometry among the alkaline-earth tellurates. Ba_3_Te_4_O_11_ is composed of [Te_8_O_22_]^12–^ corner-shared rings featuring irregular [Te_3_O_8_]^4–^ units bearing a pseudo-2-fold axis and trigonal pyramidal [TeO_3_]^2–^ units, whereas BaTeO_3_(I) and (II) are made up solely of stacked trigonal pyramidal [TeO_3_]^2–^ units. BaTeO_3_(I) is isotypic to KClO_3_ where the trigonal pyramidal [TeO_3_]^2–^ units are stacked onto each other and connected via secondary Te^4+^–O bonds along the *a*-axis. Two other monoclinic phases were synthesized hydrothermally, these being BaTe_3_O_7_ in *P*2_1_/*c* and BaTe_4_O_9_ in *C*2/*c* both of each feature a novel way of lone pair accommodation through the formation of a network of “infinite, self-contained, one-dimensional tellurite tubes”.^18^ In BaTe_3_O_7_ and BaTe_4_O_9_, the tellurite tubes are made up of [Te_3_O_7_]^2–^ and [Te_4_O_9_]^2–^ respectively, with edge-sharing tellurite moieties cross-linking adjacent tubes for the formation of a network structure. Additionally, a hydrated phase of BaTeO_3_ known as BaTeO_3_·H_2_O was originally discovered by Nielsen et al.,^19^ which consists of edge-sharing Ba^2+^ polyhedra with neighboring isolated [TeO_3_]^2–^ units. Similar to BaTeO_3_(I), it crystallizes monoclinically in the space group *P*2_1_/*a*. This structure was later reproduced hydrothermally and redetermined by Weil to reveal the position of water molecules in between the layers.^20^

The ATeO_3_ (A = Ba^2+^, Sr^2+^, or Ca^2+^) system does not adopt the perovskite structure, although its chemical formula seemingly belongs to the perovskite group. The reason in this case is the already mentioned lone pair electron at Te^4+^. BaTeO_3_,^16,17^ SrTeO_3_,^21–27^ and CaTeO_3_ ^28^ all consist of trigonal pyramidal [TeO_3_]^2–^ units but experience variation in their unit cell and motifs, with both CaTeO_3_ and SrTeO_3_ achieving a high degree of polymorphism. MgTeO_3_ is only existent as MgTeO_3_·6H_2_O crystallizing isotypically to MgSO_3_·6H_2_O.^29^ This study shows that, while HP-BaTeO_3_ retains considerable similarity to BaTeO_3_(I), it also exhibits distinct differences in its cell parameter *c*, space group, and [TeO_3_]^2–^ motif orientation. Multianvil high-pressure synthesis, structural comparison, and properties are discussed below.

## 2 Experimental Section

### 2.1 High-Pressure/High-Temperature Synthesis

BaTeO_3_(I) was first prepared as a precursor in a silica tube at 400 mbar Ar pressure according to Folger.^16^ For this step, a stoichiometric mixture of BaO (99.99%, Sigma-Aldrich, Vienna, Austria) and TeO_2_ (99.995%, Thermo Fisher Scientific, Linz, Austria) in a 1:1 ratio was ground together and placed in an alumina crucible. This crucible was then installed inside the silica tube. The setup was heated to 700 °C with a holding time of 8 h using a tube furnace assembly. In the following, the reaction mixture was cooled to room temperature with a cooling rate of 4 °C per minute. The X-ray diffraction data indicated that BaTeO_3_(I) is the main phase and only minor unidentified impurities were observed. A powder pattern can be found in Figure S1 of the SI. This precursor was then subjected to a high-pressure experiment using a 1000 t multianvil press (Walker-type module, Max Voggenreiter GmbH, Mainleus, Germany) in order to synthesize HP-BaTeO_3_. The precursor was filled into a platinum capsule (99.95%, Ögussa, Vienna, Austria), which was installed into a boron nitride crucible. An 18/11 assembly setup was used to host the sample for conducting of the high-pressure experiment, the details of which can be found in other sources.^30–32^ The sample was compressed to 4 GPa within 110 min. At this pressure, the sample was heated up until 900 °C in 10 min and held at this temperature for 3 min. The sample was then cooled to 400 °C within 30 min, followed by quenching to room temperature and decompression to ambient-pressure conditions in 330 min. The platinum capsule was extracted from the assembly, and the sample was mechanically separated from the capsule material. HP-BaTeO_3_ was obtained in an almost X-ray pure form. It appears colorless and is stable under air. Conducting the HP/HT experiment using a binary mixture of starting materials led to the formation of noticeable amounts of barium carbonate along with HP-BaTeO_3_.

### 2.2 Powder X-ray Diffraction

Powder X-ray diffraction was carried out for both ambient and high-temperature settings using a STOE Stadi P powder diffractometer (STOE & Cie GmbH, Darmstadt, Germany) equipped with a Ge(111) monochromator, a Mo K*-*L_3_ X-ray source of λ = 0.7093 Å, and a Mythen-2 1K microstrip detector (Dectris AG, Baden-Daettwil, Switzerland). A well-ground powder of HP-BaTeO_3_ was mounted in between two thin acetate foils. The powder was fixed to the acetate films by coating the latter with vacuum grease, which also ensured random crystallite orientation of HP-BaTeO_3_. Measurements were taken in transmission-geometry in the 2θ range of 2–70° with a step size of 0.015° and an exposure time of 20 s per step. A Rietveld refinement of the powder data was performed using Diffracplus-Topas 4.2 (Bruker AXS, Karlsruhe, Germany). The background of the refinement was fitted with Chebyshev polynomials of the eighth order, and the instrumental contributions were corrected through the refinement and peak shape fitting of a LaB_6_ standard.^33^ The starting model for the Rietveld refinement was derived from single-crystal data of HP-BaTeO_3_ (see Section 3.1), and the peak shape refinement was done using Thompson–Cox–Hastings pseudo-Voigt profiles.^34,35^ High-temperature powder X-ray diffraction (HT-PXRD) data were obtained by mounting a high-temperature furnace to the STOE diffractometer and installing an open 0.3 mm Mark SiO_2_ capillary within the furnace filled with a well-ground sample of HP-BaTeO_3_. The temperature was ramped up at a rate of 50 °C per minute from room temperature until 950 °C with steps of 25 °C. After each step, a powder pattern was obtained for a 2θ range of 6–24° with an acquisition time of 10 min per range.

### 2.3 Single-Crystal X-ray Diffraction

A small portion of HP-BaTeO_3_ was dispersed in a drop of perfluoropolyalkylether (viscosity 1800) placed on a microscope slide, and colorless single crystals of HP-BaTeO_3_ were selected and then mounted on the tip of MicroMounts (MiTeGen, LLC, Ithaca, NY, USA) with a loop diameter of 30 μm. Diffraction data were collected at room temperature using a Bruker D8 Quest single-crystal diffractometer (BRUKER, Billerica, USA) with Mo K-L_2,3_ radiation (λ = 0.71073 Å), an Incoatec microfocus X-ray tube (Incoatec, Geesthacht, Germany), a Photon III C14 detector system, and the Apex4 program package.^36^ Sadabs-2016/2 was used to perform the multiscan absorption correction.^37^ The structure was solved and refined using the “Intrinsic Phasing” ShelXT routine and least-squares minimization of ShelXL embedded in the Olex2 refinement program, respectively.^38–40^ The refinement was done in the space group *P*2_1_/*n*, and the space group choice was verified with Addsym routine of the Platon program package.^41^ Additionally, the Stidy routine within Platon^41^ was used for standardization of the coordinates, which were converted from *P*2_1_/*n* to standard setting *P*2_1_/*c* using the program Xprep.^42^

### 2.4 Energy-Dispersive X-ray Spectroscopy (EDX)

Semiquantitative EDX measurements were done on several crystals of HP-BaTeO_3_ using a field-emission scanning electron microscope (Clara Ultra High Resolution, TESCAN GmbH, Dortmund, Germany) equipped with an energy-dispersive Ultim Max (65 mm^2^) X-ray detector system (Oxford Instruments NanoAnalysis, Wiesbaden, Germany) for elemental identification. The crystals were mounted onto a flat aluminum sample holder with its surface fixed with adhesive carbon tape. An acceleration voltage of 20 keV and a beam current of 3 nA at a working distance of 9 mm were used for imaging and EDX data collection. The values from 12 measurement points were then averaged.

### 2.5 Infrared Spectroscopy

Infrared spectra of powdered HP-BaTeO_3_ were obtained in the 400–2500 cm^–1^ spectral range using a Bruker Alpha Platinum FTIR-ATR spectrometer (Bruker, Billerica, USA), fitted with a 2 × 2 mm diamond ATR crystal. The DTGS detector recorded intensities over 24 scans. Atmospheric effects were corrected using a reference measurement processed with Opus software.^43^

### 2.6 UV–Vis Spectroscopy

The diffuse reflectance spectra of both powdered BaTeO_3_(I) and HP-BaTeO_3_ were measured across the 250–2500 nm range using an Agilent Cary 5000 UV–vis spectrometer (Agilent, Santa Clara, United States). The device featured an integrating sphere (DRA-2500), D65 as the standard illuminant, and a 10° complementary observer. Measurements were taken with a scan rate of 600 nm/min and a data interval of 1 nm. BaSO_4_ was used as the white reference material. The Kubelka–Munk (K-M) function was applied to convert reflectance data into optical absorbance, and the bandgap was calculated using Tauc plots.^44,45^

### 2.7 Thermal Analysis

Simultaneous thermal analysis (STA) measurements (thermogravimetry/differential scanning calorimetry; TG/DSC) were conducted on a Netzsch STA 449F3 instrument (Netzsch GmbH, Selb, Germany) with an ∼37 mg sample in the temperature range of 25 ⇌ 1100 °C (corundum crucibles, a flowing argon atmosphere (50 mL min^–1^), and a heating/cooling rate of 20 °C min^–1^). A baseline correction of the TG curve was carried out by measuring the empty crucible and subtracting the data from the measurement data.

## 3 Results and Discussion

### 3.1 Composition and Structure Refinements

HP-BaTeO_3_ crystallizes in the monoclinic space group *P*2_1_/*c* with a unit cell having the following parameters: *a* = 4.5637(9), *b* = 5.976(2), *c* = 13.650(3) Å, and β = 107.3(1)°. The elemental composition was analyzed via EDX, which verified the 1:1 Ba:Te ratio of HP-BaTeO_3_ (measured Ba: 16.4 ± 2 at %, Te: 14.4 ± 2 at %). The light atom, oxygen, could not be correctly determined alongside the heavy atoms, barium and tellurium, due to the semiquantitative approach and nonideal planar crystal surfaces. No other additional elements were detected. Images of the samples and crystals as well as a table showing the composition at the individual measuring points are provided in Figures SI2–SI4 and Table SI1 in the SI. The structural and refinement data are given in Tables 1–3 and Table SI2. In addition, diffraction patterns of the 0*kl* an *hk*0 layers are depicted in Figure SI5 to demonstrate the quality of the single crystal and illustrate the agreement with the systematic absences of the chosen space group *P*2_1_/*c*. An X-ray powder diffraction pattern is presented in Figure 1. The Rietveld refinement shows a pure phase of HP-BaTeO_3_, and the refined unit-cell parameters are close to those of the single crystal (see Table 1). CSD 2465094 (HP-BaTeO_3_) contains the supplementary data for this paper.

**Table 1:** Structure Determination and Crystallographic Data of HP-BaTeO_3_ ^a^

empirical formula	BaTeO_3_
molar mass, g mol^–1^	312.94
crystal system	monoclinic
space group	*P*2_1_/*c*
cell formula units, *Z*	4
powder diffractometer	STOE Stadi P
radiation	Mo K-L_3_ (λ = 0.7093 Å)
Powder data	
*a*, Å	4.57476(8)
*b*, Å	6.0055(1)
*c*, Å	13.6728(3)
β, deg	107.371(1)
*V*, Å^3^	358.51(1)
single-crystal diffractometer	Bruker D8 Quest
radiation	Mo K-L_2,3_ (λ = 0.71073 Å)
Single-crystal data	
*a*, Å	4.5637(9)
*b*, Å	5.976(2)
*c*, Å	13.650(3)
β, deg	107.33(3)
*V*, Å^3^	355.4(2)
calculated density, g cm^–3^	5.85
crystal size, mm^3^	0.02 × 0.03 × 0.04
temperature, K	293
absorption coefficient, mm^–1^	19.03
*F*(000), e	528
detector distance, mm	38
θ range, deg	3.13–40.31
range in *hkl*	±8, ±10, ±24
total no. of reflections	17,533
data/ref parameters	2213/47
reflections with *I* > 2σ(*I*)	2211
*R*_int_/*R*_σ_	0.0468/0.0342
goodness-of-fit on *F* ^2^	1.372
absorption correction	multiscan
*R*_1_/*wR*_2_ for *I* > 2σ(*I*)	0.0205/0.0460
*R*_1_/*wR*_2_ for all data	0.0206/0.0460
extinction coefficient	0.0646(13)
transmission max./min.	0.1733/0.0774
largest diff. peak/hole, eÅ^–3^	1.396/–2.067

a Standard deviations in parentheses.

**Figure 1:**
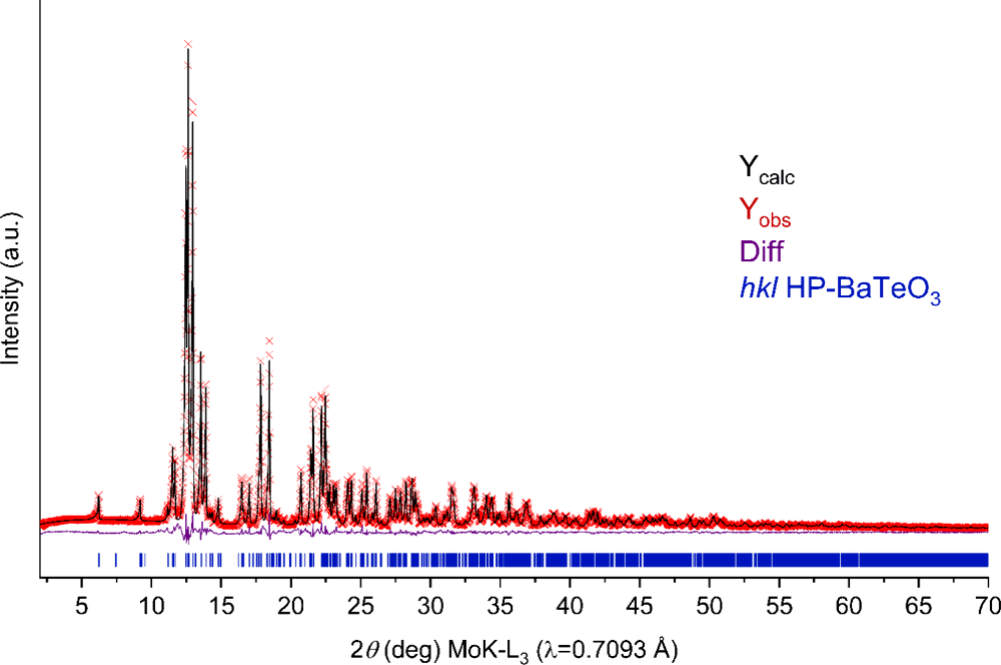
Powder X-ray diffraction pattern (Mo K-L_3_ radiation) and Rietveld refinement of HP-BaTeO_3_ (red and black curves, respectively). Reflection positions of HP-BaTeO_3_ are indicated by vertical blue bars at the bottom of the plot area (*R*_exp_ = 2.27%, *R*_wp_ = 6.27%, *R*_p_ = 4.38%, and *GooF* = 2.76).

**2:** Atomic Coordinates, Wyckoff Positions, Site Occupancy Factors (SOF), and Equivalent Isotropic Displacement Parameters *U*_eq_ (Å^2^) for HP-BaTeO_3_ (Space Group *P*2_1_/*c*; No. 14) Derived from Single-Crystal Structure Refinement^a^

atom	Wyck.	*x*	*y*	*z*	SOF	*U*_eq_
Te	4*e*	0.35967(2)	0.06377(2)	0.12423(2)	1	0.00519(4)
Ba	4*e*	0.12574(2)	0.03975(2)	0.35856(2)	1	0.00660(4)
O1	4*e*	0.0336(4)	0.5456(3)	0.3589(2)	1	0.0110(3)
O2	4*e*	0.3045(4)	0.3358(3)	0.0523(2)	1	0.0092(2)
O3	4*e*	0.5469(4)	0.1929(3)	0.2524(1)	1	0.0089(2)

a *U*_eq_ is defined as one third of the trace of the orthogonalized *U*_*ij*_ tensor (standard deviations in parentheses).

**Table 3:** Selected Interatomic Distances in HP-BaTeO_3_ Derived from Single-Crystal Structure Refinement^a^

atom	length (Å)	atom	length (Å)
Te–O1^(i)^	1.879(2)	Ba–O2^(iv)^	2.633(2)
Te–O2^(ii)^	1.877(2)	Ba–O2^(v)^	2.801(2)
Te–O3^(ii)^	1.870(2)	Ba–O2^(i)^	2.871(2)
Ba–O1^(i)^	2.838(2)	Ba–O3^(vi)^	2.764(2)
Ba–O1^(iii)^	2.983(2)	Ba–O3^(ii)^	2.880(2)
Ba–O1^(ii)^	3.053(2)	Ba–O3^(v)^	3.189(2)

a Symmetry codes: (i) −*x*, −1/2 + *y*, 1/2 – *z*; (ii) *x*, *y*, *z*; (iii) *x*, −1 + *y*, *z*; (iv) *x*, 1/2 – *y*, 1/2 + *z*; (v) 1 – *x*, −1/2 + *y*, 1/2 – *z*; (vi) −1 + *x*, *y*, *z*. Standard deviations in parentheses.

### 3.2 Crystal Chemistry

HP-BaTeO_3_ consists of one Te^4+^ site, one Ba^2+^ site, and three O^2–^ sites. It is made up entirely of trigonal pyramidal [TeO_3_]^2–^ units stacked perfectly onto each other along the *a*-axis. The Te–O bond lengths range from 1.870 to 1.879 Å, which are within the expected bond range for short Te–O bonds.^6,46^ The Ba^2+^ cation experiences a 9-fold oxygen coordination with a geometry resembling that of a distorted monocapped cube with Ba–O distances ranging from 2.632 to 3.189 Å (see Table 3). The average Ba–O bond length is 2.890 Å, which agrees well with typical values found in the literature.^47^ Apart from the primary Te–O bonds constituting the trigonal pyramidal geometry, Te^4+^ also experiences secondary bonding with oxygen atoms (typically found in crystal structures containing Te^4+^ with lone pair electrons), which consist of longer and weaker bonds compared to primary bonds.^48^ In HP-BaTeO_3_, one secondary bond of 2.739 Å occurs parallel to the *bc* plane interconnecting multiple neighboring stacked sequences of [TeO_3_]^2–^ units (see Figure 2 right) and an additional secondary bond of 2.712 Å connects the multiple stacked [TeO_3_]^2–^ units along the *a*-axis (see Figure 3 right).

**Figure 2:**
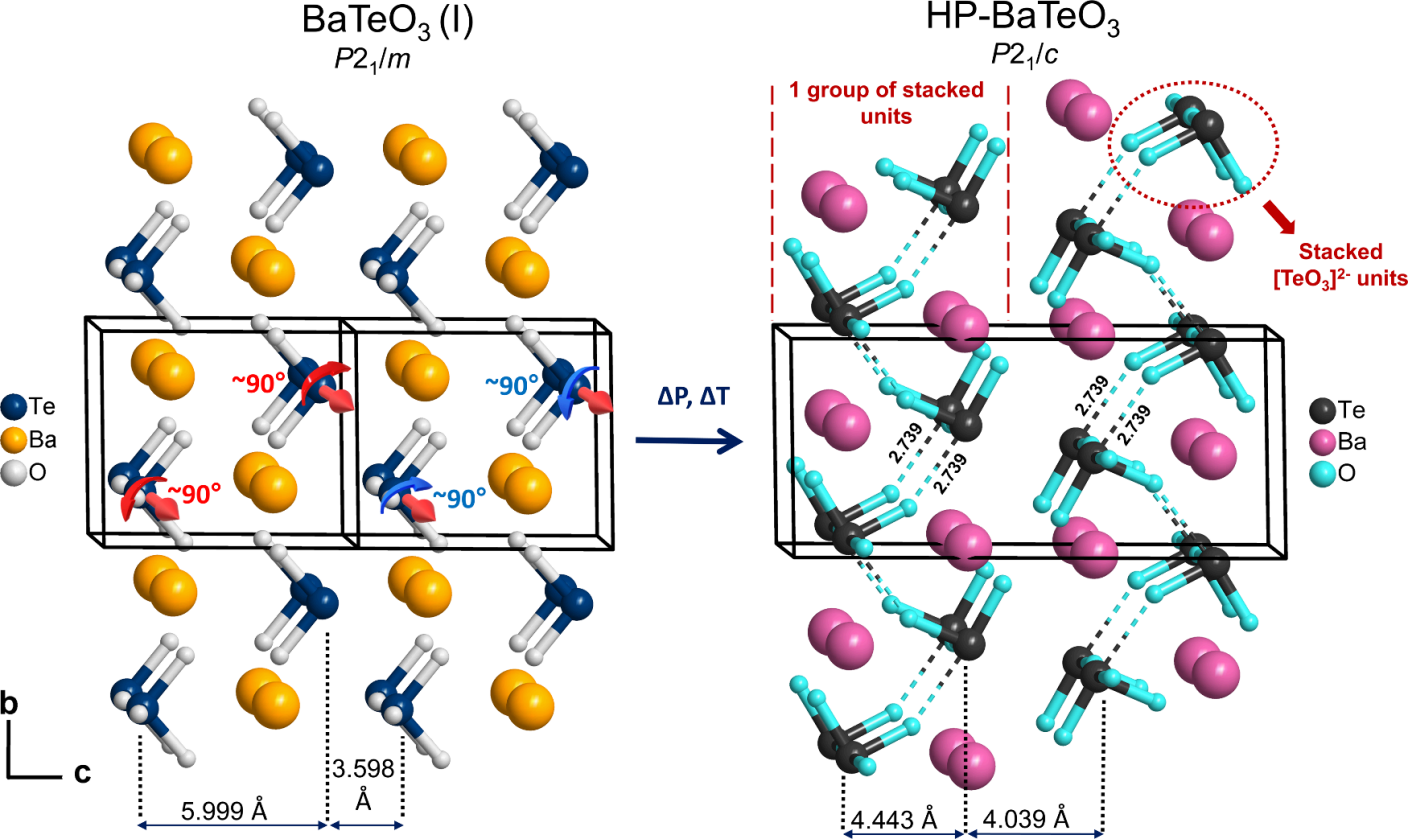
Left: Unit-cell outline of BaTeO_3_(I)^17^ showing the stacking of [TeO_3_]^2–^ units along the *a*-axis in the plane of the page. Thick red arrows show the axis of rotation of pairs of stacked [TeO_3_]^2–^ units. Rotation by approximately 90° produces a configuration similar to HP-BaTeO_3_ with red and blue arrow pairs showing the direction of rotation required. Right: Unit-cell outline of HP-BaTeO_3_ showing groups of [TeO_3_]^2–^ units stacked along the *a*-axis arranged in a corrugated manner and connected by secondary bonds parallel to the *bc* plane. Dashed lines delineate secondary bonds between [TeO_3_]^2–^ units (distances marked in Å). Double-headed arrows delineate interatomic distances parallel to the *bc* plane between neighboring Te atoms.

**Figure 3:**
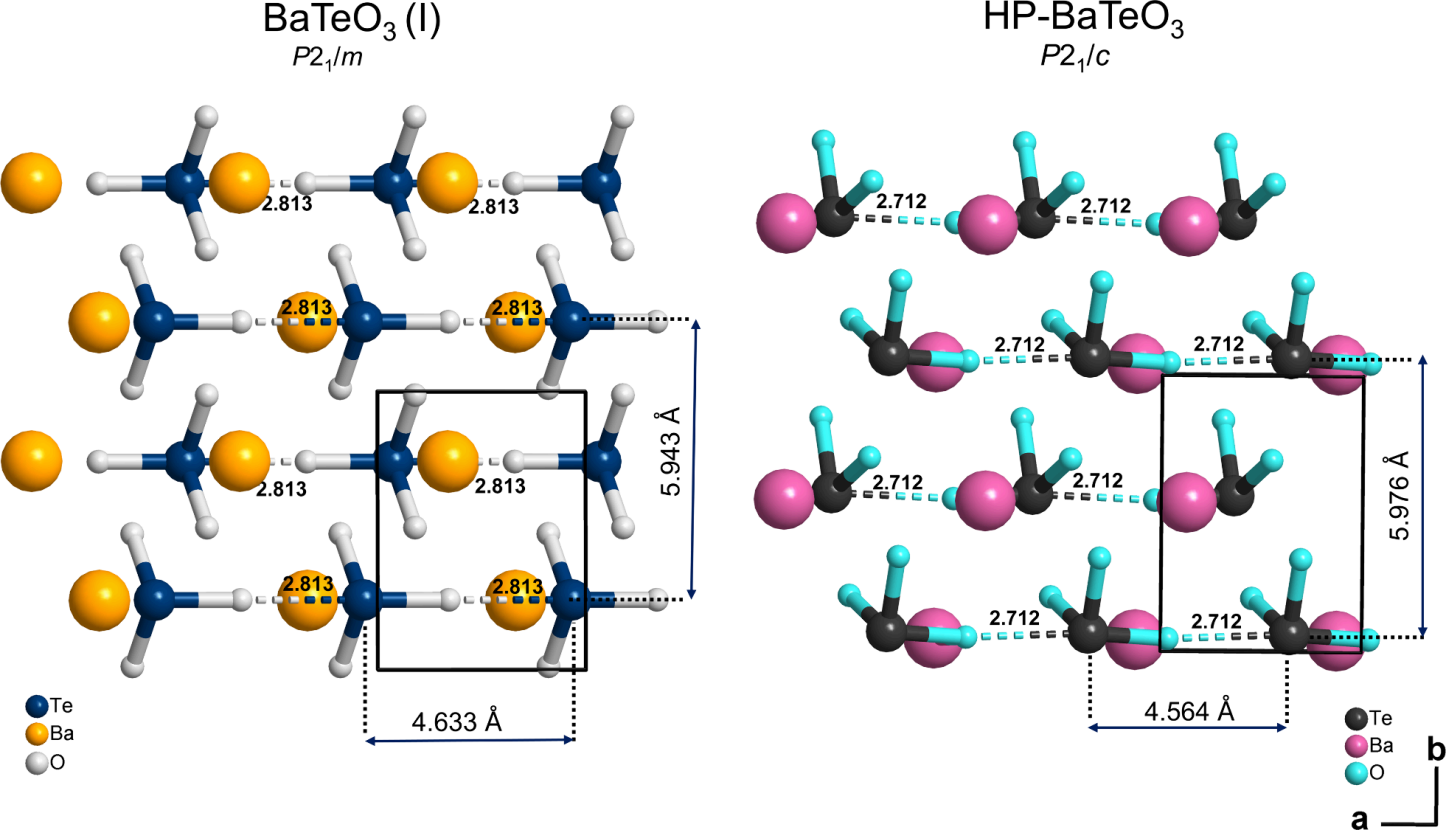
Unit-cell outline of BaTeO_3_(I)^17^ and HP-BaTeO_3_ showing the presence of secondary bonds connecting the trigonal pyramidal units along the *a*-axis in both structures. Distances separating stacked [TeO_3_]^2–^ units are marked by double-headed arrows. Only one group of [TeO_3_]^2–^ units with the associated Ba^2+^ is shown for HP-BaTeO_3_ (i.e., only half the unit cell along its *c*-axis) to allow easier visualization and comparison.

However, the stacked [TeO_3_]^2–^ sequences of HP-BaTeO_3_ are not entirely interconnected by secondary bonds throughout its structure. Instead, the structure can be viewed as groups composed of multiple sequences of stacked [TeO_3_]^2–^ units. As is shown in Figure 2, the arrangement of neighboring stacked sequences within each group is in a corrugated fashion. Ba^2+^ fills the gaps left between adjacent stacking sequences in a similar corrugated manner. Adjacent stacked groups have an antiparallel orientation of their pyramidal [TeO_3_]^2–^ units, rendering the structure centrosymmetric and canceling out polarity.

There are obvious structural relationships between the BaTeO_3_ polymorphs BaTeO_3_(I) and HP-BaTeO_3_, which will be discussed in more detail below. For BaTeO_3_(I), the crystallographic data from Koçak et al. are always used for this comparison.^17^ Both compounds are similar in that they are entirely made up of stacked trigonal pyramidal [TeO_3_]^2–^ units and have related space groups and their *a* and *b* lattice parameters are similar, but the *c* parameter is doubled in HP-BaTeO_3_ (see Table 4).

**Table 4:** Comparison of Cell Parameters, Charge Distribution (Calculated with Bond Length/Bond Strength, BL/BS (∑*V*), and Chardi (∑*Q*)) and Maple Values of Both HP-BaTeO_3_ and BaTeO_3_(I)^17^

			BL/BS and Chardi valences					
	cell parameter (Å)		Te	Ba	O1	O2	O3	Maple (kJ/mol)
HP-BaTeO_3_	*a* = 4.564	Σ*V*	+4.00	+1.91	–1.82	–1.93	–1.89	15,803.6
*P*2_1_/*c*	*b* = 5.976	Σ*Q*	+4.02	+1.98	–1.87	–2.18	–1.95	
	*c* = 13.650							
	β = 107.3°							
BaTeO_3_(I)^17^	*a* = 4.633	Σ*V*	+3.94	+1.76	–1.73	–1.99		15,417.1
*P*2_1_/*m*	*b* = 5.943	Σ*Q*	+4.06	+1.95	–1.99	–2.01		
	*c* = 7.104							
	β = 106.4°							

The similarity in these lattice parameters can be rationalized by considering that HP-BaTeO_3_ and BaTeO_3_(I) show very similar distances between [TeO_3_]^2–^ units along both the *a-* and *b*-axes (see marked Te–Te distances in Figure 3). Furthermore, as shown in Figure 2, both structures retain the corrugated positioning of the Te^4+^ parallel to the *bc* plane. Hence, the structures are mostly different along the *c*-axis, which is approximately double for HP-BaTeO_3_ relative to that of BaTeO_3_(I). Figure 2 shows that BaTeO_3_(I) is related to HP-BaTeO_3_ via rotation of the trigonal pyramidal units by approximately 90° in opposite directions around an axis parallel to the *a*-axis. The antiparallel orientation of adjacent groups of [TeO_3_]^2–^ units requires adjacent unit pairs to consecutively rotate toward and away from each other (see Figure 2: red and blue arrow pairs). Due to this antiparallel orientation, translational symmetry of BaTeO_3_(I) along the *c*-axis is broken, hence requiring a doubling of this axis. These described structural relationships suggest a group–subgroup relationship and an associated displacive phase transition of higher order between the two structures. The space group *P*2_1_/*c* is a maximal klassengleiche subgroup of *P*2_1_/*m* with index 2. In this context, a basis transformation is also necessary, which corresponds to our observed doubling of the *c*-axis. The 4*f* Wyckoff site of the O2 atom of BaTeO_3_(I) would split into two 4*e* sites, which correspond to the atom sites O2 and O3 of HP-BaTeO_3_. After taking into account a shift of the unit cell of approximately −1/2*a*, 1/4*b*, *c*, it is possible to get the Ba atoms to comparable atom sites. A clear visualization can be found in Figures SI6 and SI7 in the SI, which shows a superposition of the two crystal structures. However, the Te atom positions show larger deviations and a site continuation for the oxygen atoms is no longer ensured. This can be attributed to the different oriented 90° rotation of the [TeO_3_]^2–^ units in HP-BaTeO_3_. Compared to BaTeO_3_(I), [TeO_3_]^2–^ units of HP-BaTeO_3_ experience twice the secondary bonding with the additional secondary bonds occurring parallel to the *bc* plane connecting neighboring lines of stacked [TeO_3_]^2–^ units. This feature is absent in BaTeO_3_(I) where secondary bonds occur only in the stacking (*a*) direction. The Te–Te distances along the *c* direction also experience variation between the two phases, unlike those along the *a* and *b* directions. BaTeO_3_(I) has a wide difference in its Te–Te distances standing at 3.598 and 5.999 Å, compared to those of HP-BaTeO_3_, which stand closer together at 4.039 and 4.442 Å. Figure 3 compares the two structures along their similar *a*- and *b*-axes. Here, the near identical stacking arrangements of the trigonal pyramids of both structures are clearly shown, but a slight relative displacement of neighboring chains is also apparent. This is accompanied by minor shortening of the Te–Te distances in both the *a* and *b* directions as BaTeO_3_(I) is converted to HP-BaTeO_3_. HP-BaTeO_3_ achieves a 6.2% higher density relative to that of BaTeO_3_(I) (ρ_BaTeO3(I)_ = 5.51 g cm^–3^), which is a common feature for high-pressure phases compared to their ambient counterparts. These considerable structural changes force a reconstructive phase transition of the first order that can no longer be described by a group–subgroup relationship.

A search in the ICSD crystallographic database^49^ against Wyckoff sequence *e* ^5^ gave crystal structures containing other lone pair cations with a similar structure to that of HP-BaTeO_3_, most notably AsSbO_3_ ^50^ and CsSnF_3_ ^51^ both of which crystallize in the space group *P*2_1_/*n*, which is the nonstandard setting of *P2*_1_/*c*. These structures feature the same groups of corrugated arrangements of stacked trigonal pyramidal units with adjacent groups arranged in an antiparallel fashion. The electron lone pairs of Sn^2+^ in CsSnF_3_ were determined to lie in channels between adjacent groups, and the lone pairs from adjacent groups have a similar antiparallel orientation, thus canceling out local polarity of the units. Both compounds have comparable unit-cell lengths, but the *β* angles vary considerably (HP-BaTeO_3_: *β* = 107.3°, AsSbO_3_: *β* = 95,0°, CsSnF_3_: *β* = 91.0°. Therefore, AsSbO_3_ and CsSnF_3_ can be classified as isostructural to HP-BaTeO_3_ not isotypic.

To verify the structure refinement of HP-BaTeO_3_, Maple (Madelung Part of Lattice Energy),^52–54^ Chardi (Charge Distribution),^55^ and BL/BS (bond length/bond strength)^46,56,57^ calculations from single-crystal data were carried out. The calculated charge distribution and bond-valence sums are shown in Table 4 for both HP-BaTeO_3_ and BaTeO_3_(I). The derived BL/BS sums and Chardi output values of both HP-BaTeO_3_ and BaTeO_3_(I) agree well with the expected formal charges of Te^4+^, Ba^2+^, and O^2–^. The Maple values for both phases are relatively similar and agree with the summation of Maple values of the starting materials (details are given in the SI) with HP-BaTeO_3_ and BaTeO_3_(I) having 0.77% and 3.2% differences, respectively.

### 3.3 Thermal Analysis

In order to further investigate the relationship between HP-BaTeO_3_ and BaTeO_3_(I), high-temperature powder X-ray diffraction (HT-PXRD) and simultaneous thermal analysis (STA) were carried out to determine the nature of the transition between the two phases. HP phases are known to be metastable; hence, they are expected to transform to their thermodynamically stable forms when supplied with enough thermal energy. The transformation between HP-BaTeO_3_ to BaTeO_3_(I) occurs completely at a temperature of about 550 °C (see Figure. [Fig fig4]; equivalent waterfall plot for the HT-PXRD is available in the SI) and suggests a first-order phase transition due to the absence of a continuous gradual transformation of the reflections of HP-BaTeO_3_ to the positions of BaTeO_3_(I). The phase BaTeO_3_(I) was confirmed through a Rietveld refinement of a single PXRD range collected at a temperature of 630 °C. Reference can be made to Figure SI10.

**Figure 4:**
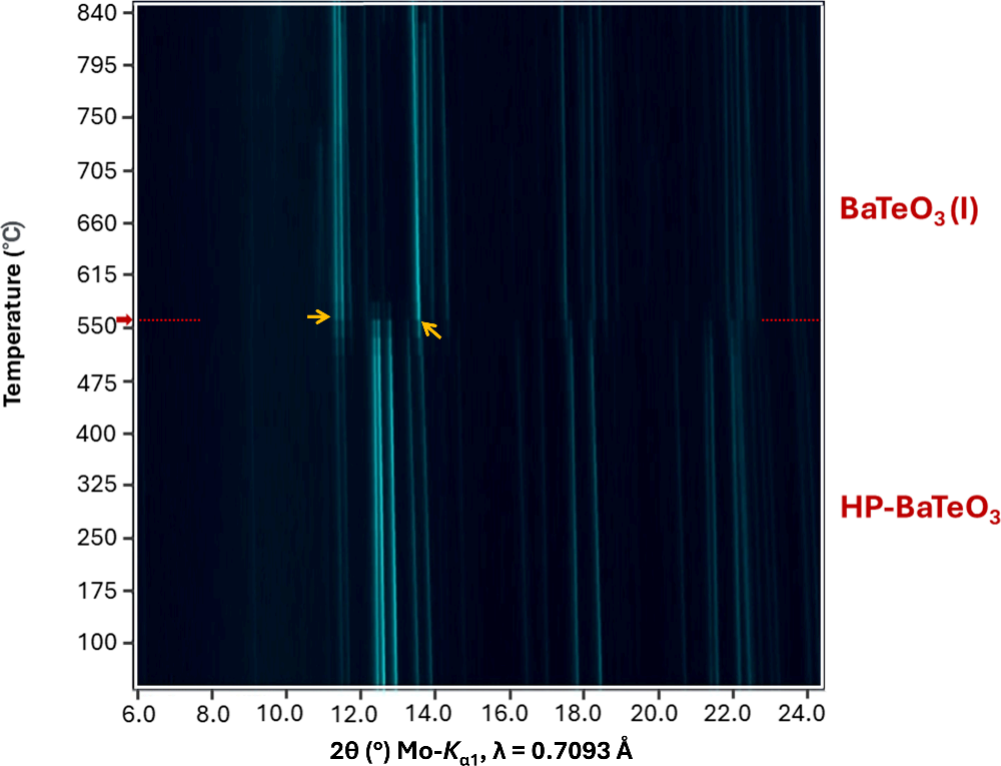
HT-PXRD plot showing the phase transition of HP-BaTeO_3_ (*P*2_1_ */c*) to BaTeO_3_(I) (*P*2_1_ */m*)^17^ at a temperature of around 550 °C. Red arrows pointing toward red dotted lines indicate the point at which most of the HP-BaTeO_3_ transforms. Yellow arrows denote similar reflections that occur in both phases.

At 550 °C, a sharp change in the pattern can be seen and only a few reflections (at 2θ = 12.5°, 12.7°, and 13.9°) show a continuous shift toward the positions of the BaTeO_3_(I) reflections. The parallel appearance of reflections that show a continuous transition to the normal pressure phase alongside reflections that disappear completely and appear at around 550 °C is also evidence of the high structural similarity of the two polymorphic structures. Ultimately, only a rotation of the [TeO_3_]^2–^ units takes place and the Ba atoms remain almost in their positions. In addition, the presence of a higher-order phase transition would necessarily require a correlation via a group–subgroup relationship between the two crystal structures, which was excluded by the group–subgroup analysis performed in Section 3.2. At temperatures higher than 855 °C, discrete reflections vanish completely, which indicates melting. Differential scanning calorimetry and thermogravimetry were also carried out to further confirm this outcome (see Figure SI8). From HT-PXRD, a DSC signal at around 550 °C was expected, which was not observable. Despite the relatively large sample quantity, neither an exothermic nor an endothermic conversion signal could be observed. The energy released during the phase transformation, caused purely by rotation of the [TeO_3_]^2–^ groups, is presumably too low to be registered in the comparatively insensitive corundum crucibles. More sensitive platinum crucibles cannot be used due to the tellurium content. After the experiment, the ambient-pressure polymorph BaTeO_3_(I) is present, confirmed by PXRD measurement. The endothermic melting signal at 997 °C is therefore assigned to the phase BaTeO_3_(I). In the temperature range around 650–800 °C, a mass loss of approximately 1% can also be observed, which is possibly a consequence of the phase transformation with accompanied decomposition.

### 3.4 IR Spectroscopy

Both the IR spectra of HP-BaTeO_3_ and its precursor BaTeO_3_(I) were obtained and are presented in Figure 5. The IR spectrum of BaTeO_3_(I) was already investigated by Arnaudov et al.^58^ revealing two bands at 750 and 680 cm^–1^ belonging to the symmetric (*v*_1_) and asymmetric (*v*_3_) Te–O stretch vibrations, respectively. In this study, it is additionally shown that the *v*_3_ band is split into two bands at 660 and 674 cm^–1^, with the latter occurring as a shoulder band to the former band having the strongest intensity. This splitting of the *v*_3_ band can be attributed to crystal field effects decreasing the force constant of the Te–O bond. Due to the similarity in structure and composition, the IR spectrum of HP-BaTeO_3_ shows a similar pattern of absorption bands lying at 629, 652, and 728 cm^–1^. Therefore, similar to BaTeO_3_(I), the absorption at 728 cm^–1^ can be assigned to the *v*_1_ mode whereas 629 and 652 cm^–1^ are both assigned to a splitting of the *v*_3_ mode, with the former band showing the highest intensity. Some shift to lower wavenumbers is therefore observed for HP-BaTeO_3_ relative to those of BaTeO_3_(I). Compared to BaTeO_3_(I), the IR spectrum of HP-BaTeO_3_ also shows an additional band lying at 495 cm^–1^, which is here attributed to the *v*_2_ mode consisting of Te–O bending vibrations.

**Figure 5:**
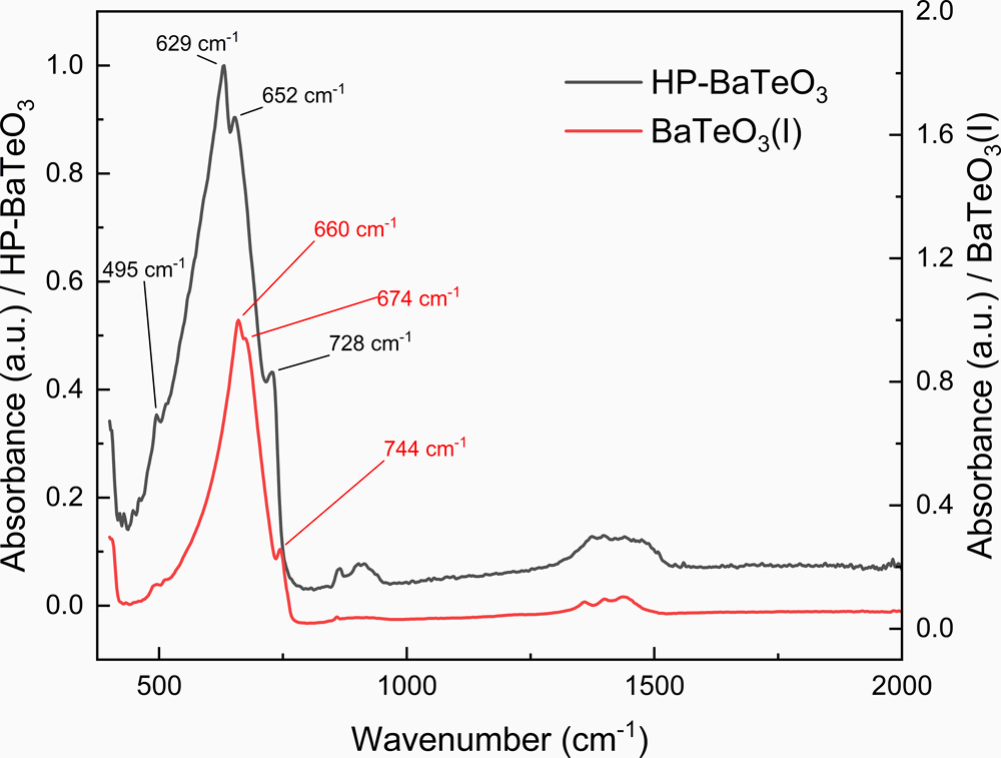
Infrared spectra of HP-BaTeO_3_ and BaTeO_3_(I) (from our own precursor synthesis).

### 3.5 UV–Visible Spectroscopy

Diffuse reflectance UV–vis spectra for BaTeO_3_(I) and HP-BaTeO_3_ in the range of 250–2500 nm are presented in Figure 6 (top). The absorption edge of HP-BaTeO_3_ is clearly shifted toward shorter wavelengths and higher energy. This can also be seen in the energies for the direct and indirect bandgaps determined using the Kubelka–Munk^45^ function and Tauc plots.^44^ The optical bandgaps for BaTeO_3_(I) and HP-BaTeO_3_ were determined to be *E*_g(direct)_ = 3.8 eV, *E*_g(indirect)_ = 3.7 eV and *E*_g(direct)_ = 4.3 eV, *E*_g(indirect)_ = 4.2 eV, respectively (see Figure 6 (bottom)). Pressure-induced bandgap tuning is a well-known phenomenon in materials chemistry and is related to changes in the electronic structure. The main difference in the crystal structures of the two BaTeO_3_ polymorphs is the rotation of the [TeO_3_]^2–^ groups and thus also the different orientation of the lone pair electrons. As a result, HP-BaTeO_3_ shows an additional secondary bonding option as described. This structural change is expected to be the main reason for the widening of the bandgap. Theoretical DFT calculations to explain the phenomenon are planned. Both the widening and the narrowing of the bandgap can be observed under increasing pressure conditions, although the latter is much more common.^59,60^ In many cases, bandgap widening is associated with pressure-induced disorder in materials.^61^

**Figure 6:**
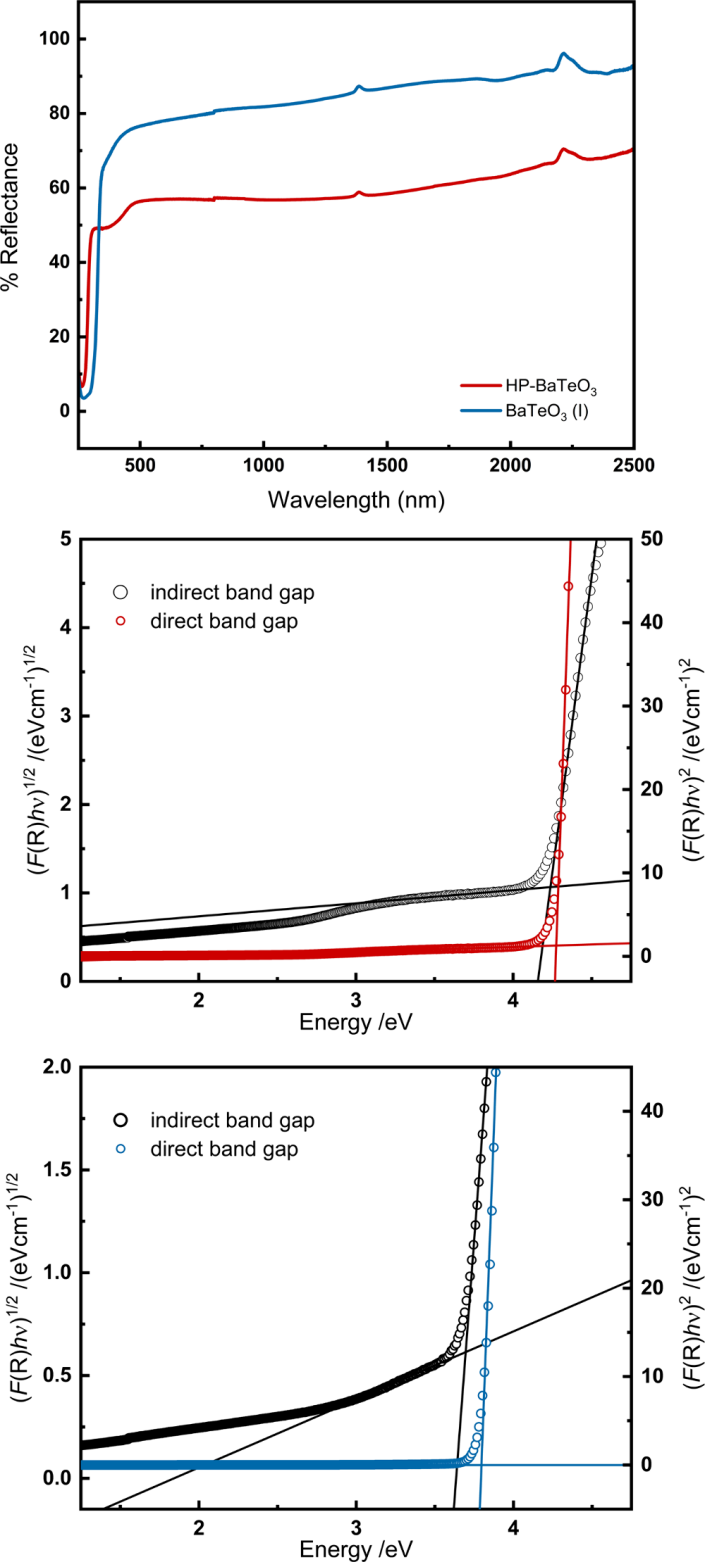
(Top) UV–visible spectra of HP-BaTeO_3_ and BaTeO_3_(I). Tauc plots of HP-BaTeO_3_ (middle) and BaTeO_3_(I) (bottom) to estimate the direct (4.3 and 3.8 eV) and indirect (4.2 and 3.7 eV) bandgaps.

## 4 Conclusions

The high-pressure phase HP-BaTeO_3_ was successfully synthesized and characterized, revealing significant structural and electronic differences compared to its ambient-pressure counterpart, BaTeO_3_(I). HP-BaTeO_3_ crystallizes in the monoclinic space group *P*2_1_/*c*, with a unit cell approximately double the *c*-axis of BaTeO_3_(I). The structure features additional secondary bonding within the *bc* plane, which contributes to its increased density and altered electronic properties. UV–vis spectroscopy demonstrated a widened bandgap for HP-BaTeO_3_, attributed to changes in orbital overlap and lone pair orientation. Thermal analysis and high-temperature X-ray diffraction confirmed the metastable nature of HP-BaTeO_3_, with a reconstructive phase transition to BaTeO_3_(I) at 550 °C. Group–subgroup analyses exclude a direct connection between the two polymorphs via a higher-order phase transformation. These findings underscore the impact of high-pressure synthesis on the structural and electronic properties of barium tellurates and provide a foundation for further exploration of high-pressure phases in related systems.

## Supplementary Material



## Notes

Powder XRD of BaTeO_3_(I), electron micrographs, single-crystal diffraction images, structure overlay of BaTeO_3_(I) and HP-BaTeO_3_, DSC and TGA plots, waterfall plot of HT-PXRD measurement, Rietveld plot of BaTeO_3_(I), MAPLE calculation, additional tables of EDX data, and anisotropic displacement parameters for HP-BaTeO_3_ (PDF)
