# Supplementary material for: Polymorphism of BaTeO3 under High Pressure: Single-Crystal Structure Analysis and Characterization of HP-BaTeO3
Source: Cryst Growth Des. 2026 Jan 22;26(3):1253–61. doi: 10.1021/acs.cgd.5c01378 (PMC12879537; doi:10.1021/acs.cgd.5c01378)
Supplement: Supplementary file 1 [file cg5c01378_si_001.pdf]

Electronic Supporting Information

Polymorphism of $BaTeO_3$ under high pressure: single-crystal structure analysis and characterisation of HP-$BaTeO_3$

Benjamin J. Pullicino, Stefan Schwarzmüller, and Gunter Heymann*

*Institut für Allgemeine, Anorganische und Theoretische Chemie, Universität Innsbruck, Innrain 80-82, 6020 Innsbruck, Austria

Table of contents

1. Figures

Figure SI1. Powder XRD of the starting precursor $BaTeO_3$(I)
Figure SI2. Electron micrographs of HP-$BaTeO_3$
Figure SI3. Electron micrographs of HP-$BaTeO_3$
Figure SI4. Electron micrographs of HP-$BaTeO_3$
Figure SI5 Diffraction image of the 0*kl* and *hk*0 layer
Figure SI6. Structure overlay of $BaTeO_3$(I) and HP-$BaTeO_3$ – *ab* plane
Figure SI7. Structure overlay of $BaTeO_3$(I) and HP-$BaTeO_3$ – *bc* plane
Figure SI8. DSC and TGA plots for a sample of $BaTeO_3$
Figure SI9. Waterfall plot for HP-$BaTeO_3$ HT-PXRD measurement
Figure SI10. Rietveld plot of $BaTeO_3$(I) after phase transition

2. Tables

Table SI1. EDX data of HP-$BaTeO_3$
Table SI2. Anisotropic displacement parameters for HP-$BaTeO_3$

1. MAPLE calculations (Madelung Part of Lattice Energy)

In lattice energy calculations, the Madelung part involves the Madelung constant (A), which is a dimensionless factor accounting for all long-range electrostatic interactions between ions in a crystal lattice. It also involves the Born–Landé equation, which combines this constant with repulsive forces in order to estimate the total lattice energy. The Madelung constant depends on the specific crystal structure, and it is found by summing alternating positive and negative electrostatic terms for ions at increasing distances from a central ion.

$MAPLE_{ter}$ = *MAPLE* value obtained for HP-$BaTeO_3$ or $BaTeO_3$(I).

$MAPLE_{\mathrm{ter}}$ (HP-$BaTeO_3$) = 15803.56 $kJmol^{-1}$

$MAPLE_{\mathrm{ter}}$ ($BaTeO_3$(I)) = 15417.08 $kJmol^{-1}$

$MAPLE_{\mathrm{bin}}$ = *MAPLE* value obtained for summation of individual *MAPLE* values for binary components BaO and TeO_2 used to produce both HP-$BaTeO_3$ and $BaTeO_3$(I).

$MAPLE_{\mathrm{bin}}$ = *MAPLE* (BaO) + *MAPLE* (TeO_2) = 3519.72 $kJmol^{-1}$ + 12406.97 $kJmol^{-1}$
= 15926.69 $kJmol^{-1}$

$MAPLE_{\Delta} = MAPLE_{\mathrm{bin}} - MAPLE_{\mathrm{ter}}$

$MAPLE_{\Delta}$(HP-$BaTeO_3$) = 15926.69 $kJmol^{-1}$ - 15803.59 $kJmol^{-1}$ = 123.1 $kJmol^{-1}$

123.1 $kJmol^{-1}$ / 15926.69 kJmol-1 × 100 = **0.77%**

$MAPLE_{\Delta}$($BaTeO_3$(I)) = 15926.69 $kJmol^{-1}$ - 15417.08 $kJmol^{-1}$ = 509.61 $kJmol^{-1}$

509.61 $kJmol^{-1}$ / 15926.69 kJmol-1 × 100 = **3.2%**

2. Figures

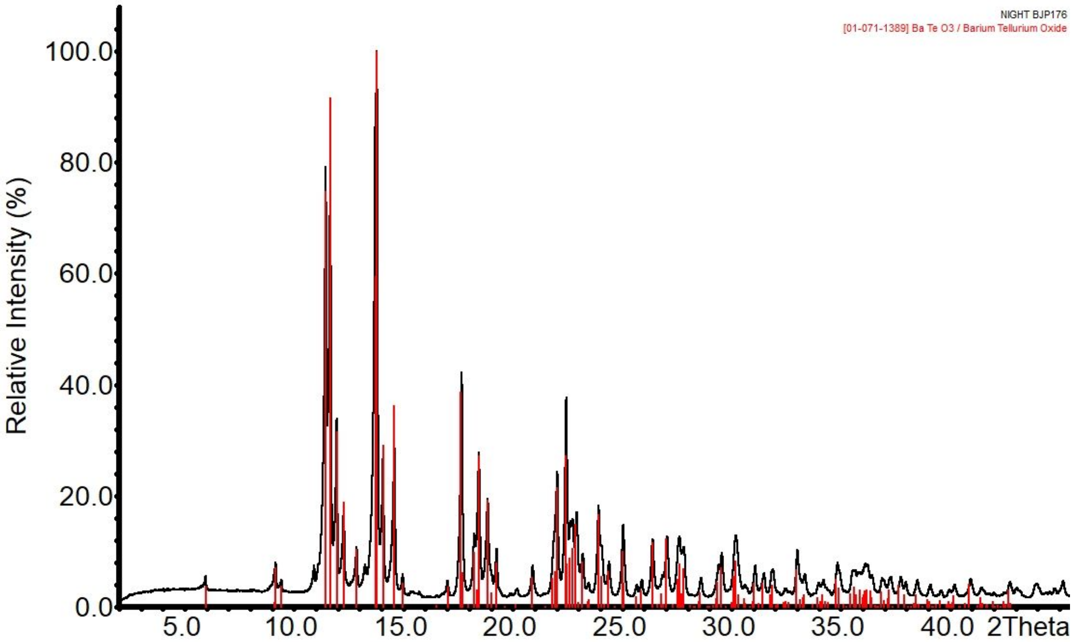

Figure SI1. Powder XRD of the starting precursor $BaTeO_3$(I) (black) with the underlying reflection positions (red).

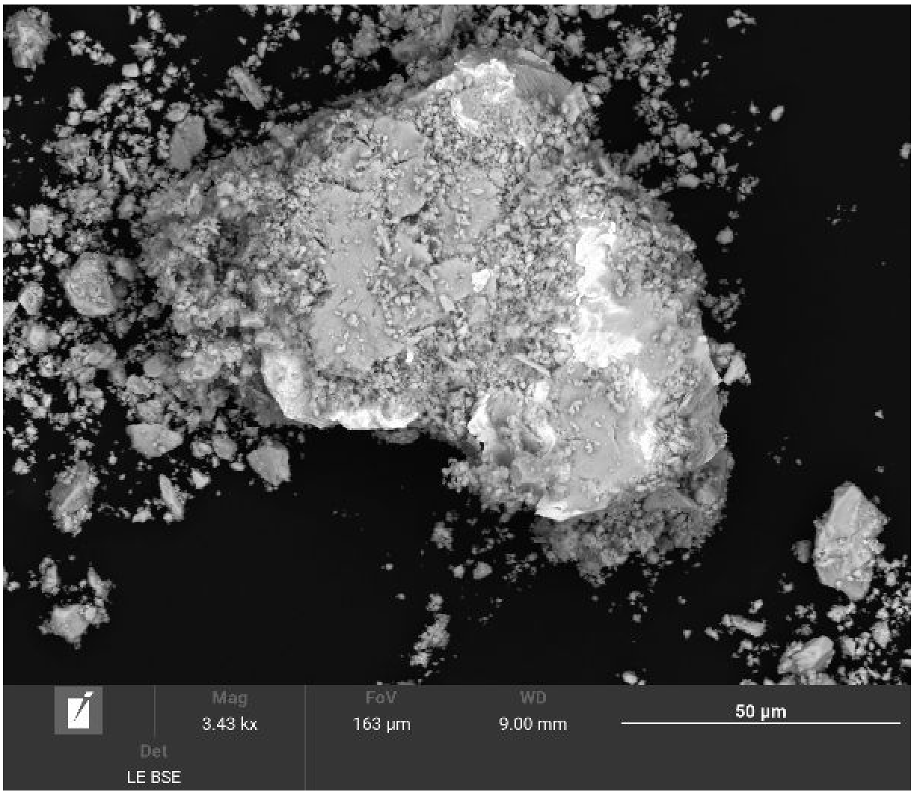

Figure SI2. Electron micrograph of HP-$BaTeO_3$.

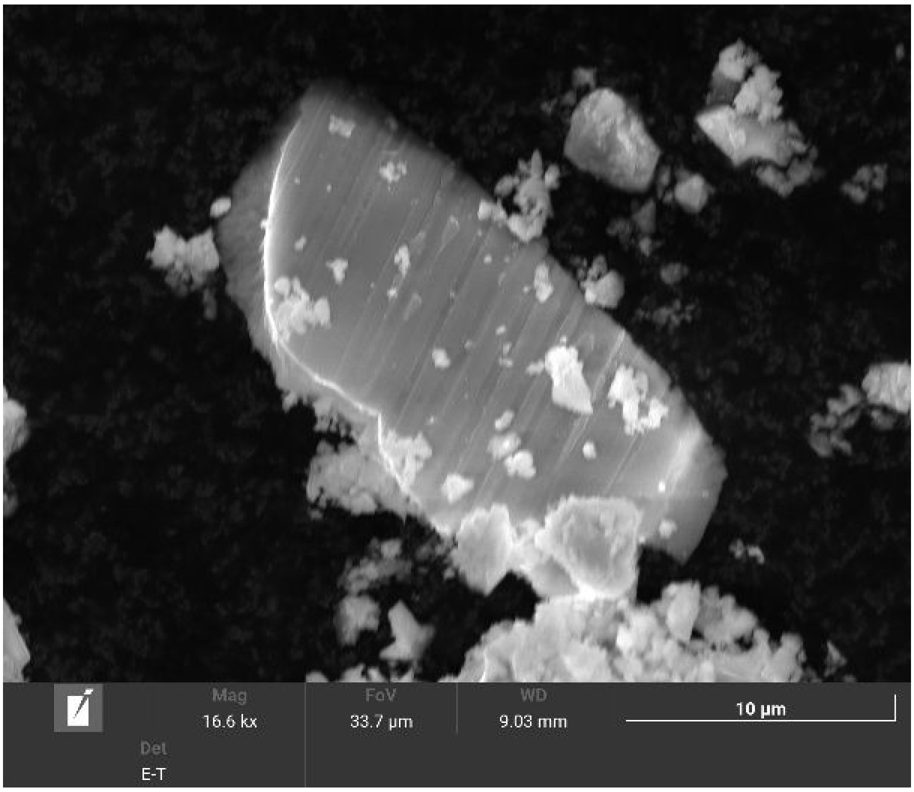

Figure SI3. Electron micrograph of HP-$BaTeO_3$.

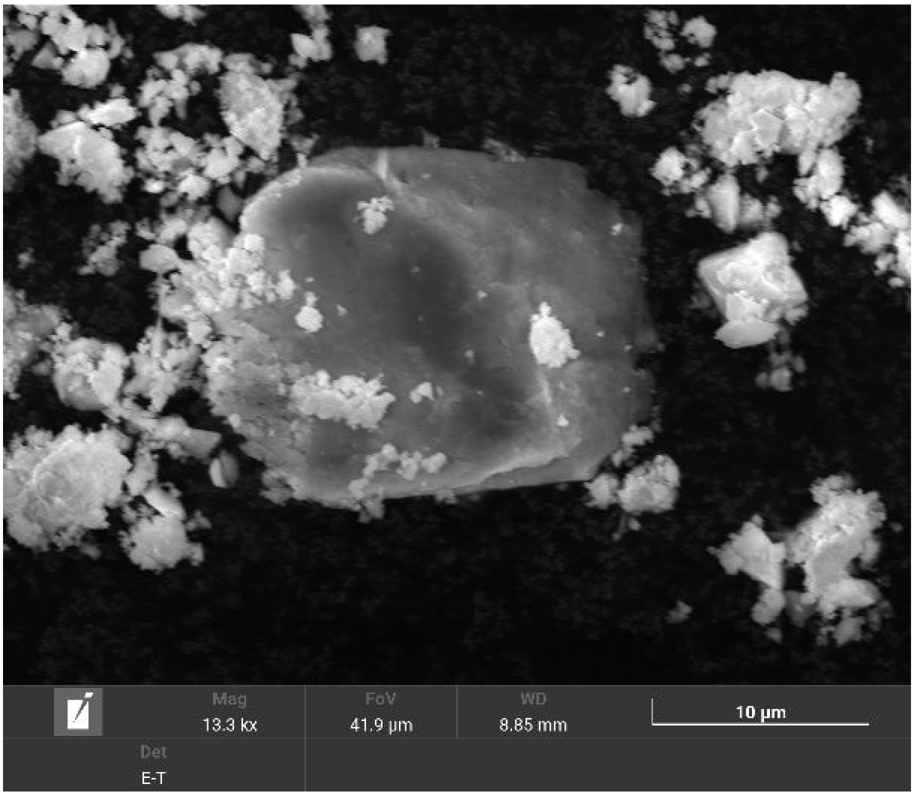

Figure SI4. Electron micrograph of HP-$BaTeO_3$.

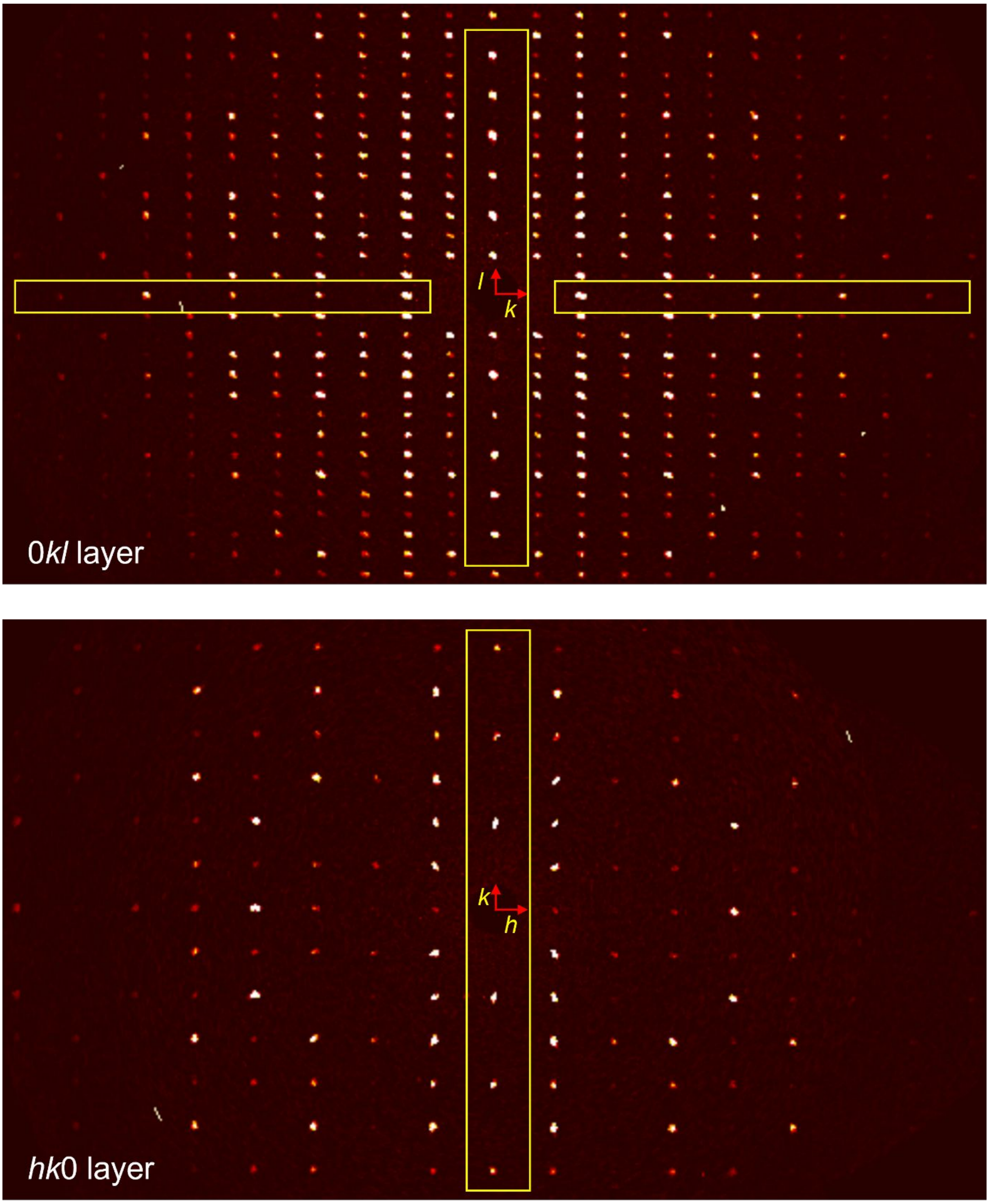

Figure SI5. (top)Reflection conditions 00*l*: *l*=2*n* and 0*k*0: *k*=2*n* can be identified, (bottom) reflection condition 0*k*0: *k*=2*n* can be identified.

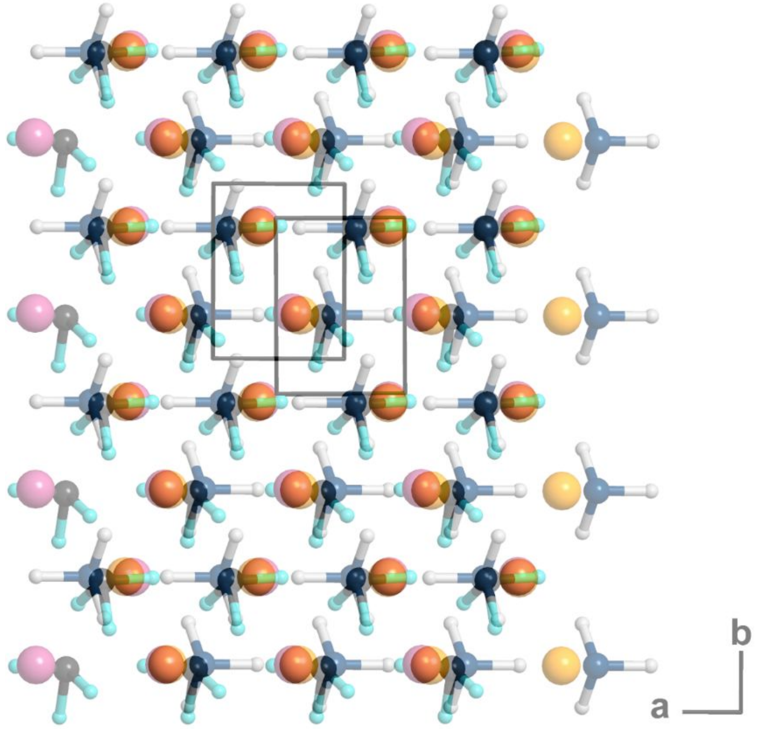

Figure SI6. Overlay of the unit cell of $BaTeO_3$(I) and half of the unit cell of HP-$BaTeO_3$ parallel to the *ab* plane.

The overlay shows that the structure of HP-$BaTeO_3$ and $BaTeO_3$(I) coincide in terms of both their Ba^{2+} and Te^{4+} positions along the *ab* plane when an origin shift of approximately ½ *a* and ¼ *b* is applied. For HP-$BaTeO_3$ this applies to **only one half** of the unit cell, as shown in this figure, as the other half is displaced relative to the half shown here. This will be clearly shown in Figure SI5.

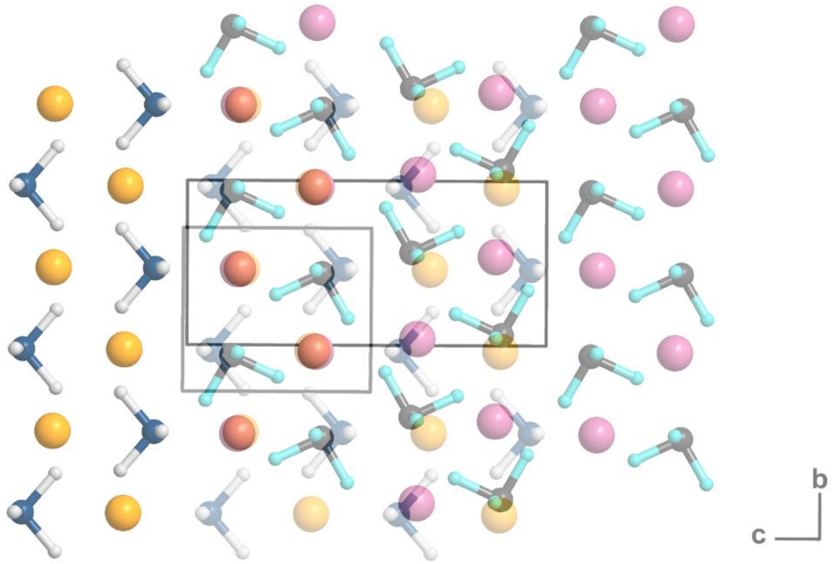

Figure SI7. Overlay of the unit cell of $BaTeO_3$(I) and half of the unit cell of HP-$BaTeO_3$ parallel to the *bc* plane.

The overlay shows coincidence also along the *c* direction of the Ba^{2+} of $BaTeO_3$(I) and the left half the unit cell of HP-$BaTeO_3$ when the origin is moved by ½ *a* and ¼ *b*. For the other right half of the unit cell of HP-$BaTeO_3$, the Ba^{2+} do not match those of $BaTeO_3$(I) because the (½ *a*, ¼ *b*) shift must go in the opposite direction of the left half due to the centrosymmetry of the structure. For Te^{4+} both halves of HP-$BaTeO_3$ do not coincide well with those of $BaTeO_3$(I) but more so for the right half (where the Te^{4+} atoms of both structures are shown to differ greatly) for the same centrosymmetry reasons.

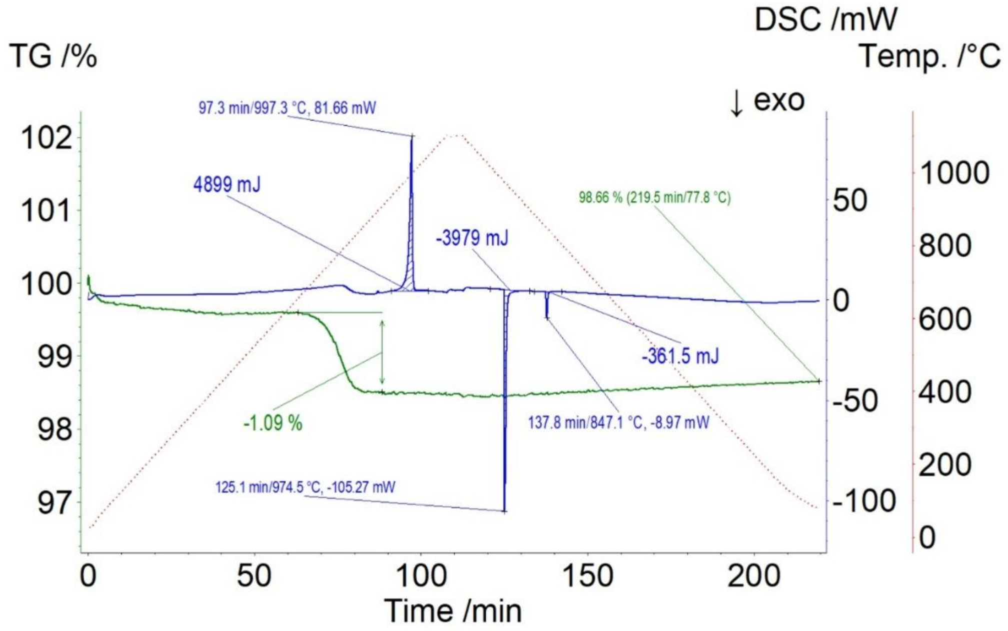

Figure SI8. DSC and TGA plots for a sample of HP-$BaTeO_3$

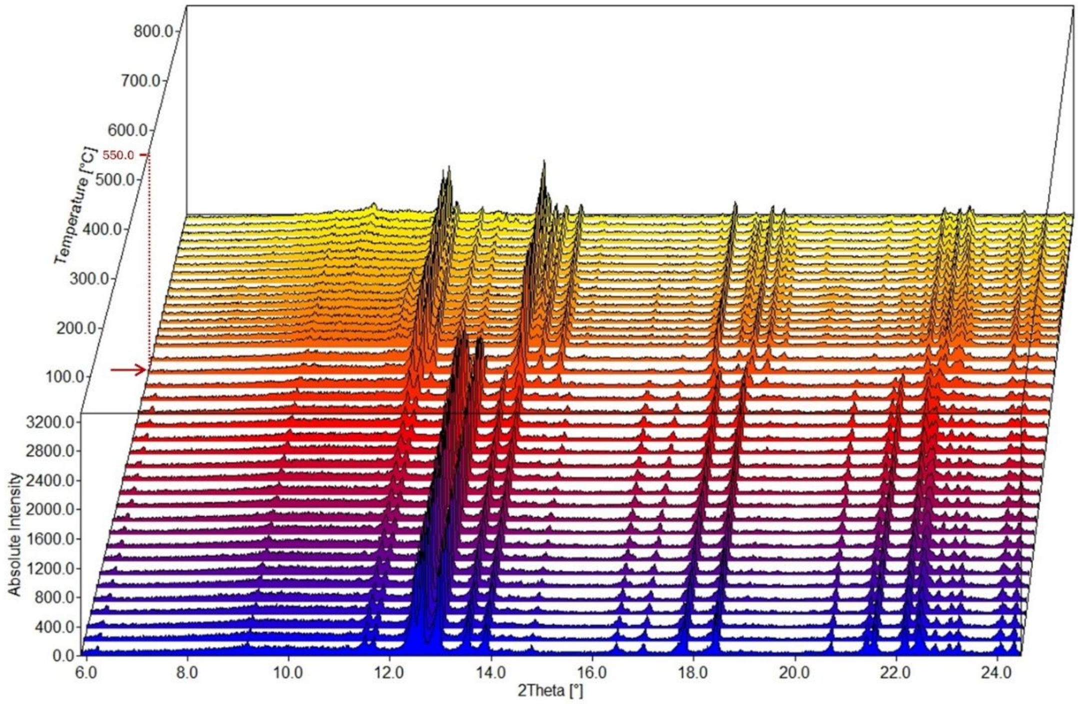

Figure SI9. Waterfall plot equivalent of HT-PXRD of HP-$BaTeO_3$. The red arrow marks the phase transition from HP-$BaTeO_3$ to $BaTeO_3$(I) at around 550 °C.

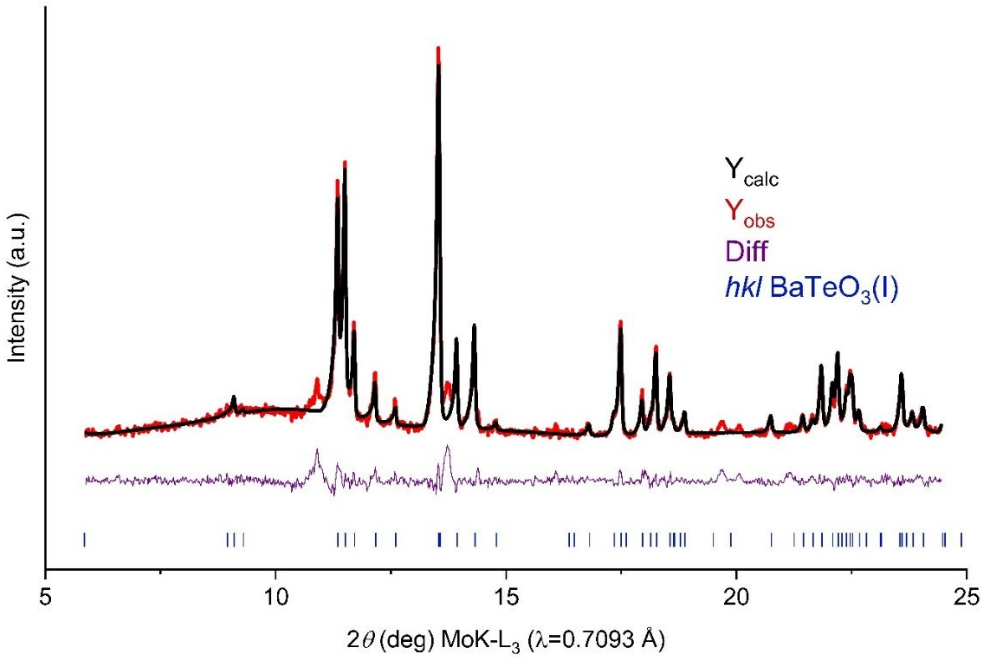

Figure SI10. Rietveld refinement of PXRD at 630 °C showing presence of $BaTeO_3$(I) as the main phase after phase transition from HP-$BaTeO_3$ at high temperature. (R_{exp} = 8.97%, R_{wp} = 12.05%, R_p = 8.43%, *GooF* = 1.34). Lattice parameters obtained after refinement (a = 4.6650(3) Å, b = 6.0034(5) Å, c = 7.2572(6) Å, β = 106.705(6)°) are slightly larger than those found in the literature for $BaTeO_3$(I) due to temperature-induced unit cell expansion. Additional reflections are presumably decomposition products that could not be identified in detail.

3. Tables

Table SI1. EDX data of HP-$BaTeO_3$

**Number**	**O**	**Te**	**Ba**	**Total**
1	69.59	14.32	16.09	100
2	68.84	14.87	16.29	100
3	68.51	14.86	16.63	100
4	68.94	14.62	16.44	100
5	68.82	14.34	16.84	100
6	70.67	13.7	15.63	100
7	68.82	14.7	16.48	100
8	70.28	14.01	15.71	100
9	68.87	14.8	16.33	100
10	68.25	14.8	16.95	100
11	68.23	14.78	16.99	100
12	70.08	13.08	16.83	100

**Statistic**	**O**	**Te**	**Ba**
**Max**	70.67	14.87	16.99
**Min**	68.23	13.08	15.63
**Average**	69.16	14.41	16.43
**Standard Deviation**	0.81	0.56	0.45

Table SI2. Anisotropic displacement parameters U_{ij} (Å^2) for HP-$BaTeO_3$ (space group $P2_1/c$; no. 14) derived from single-crystal structure refinement. Standard deviations are provided in parentheses.

**Atom**	$\boldsymbol{U_{11}}$	$\boldsymbol{U_{22}}$	$\boldsymbol{U_{33}}$	$\boldsymbol{U_{23}}$	$\boldsymbol{U_{13}}$	$\boldsymbol{U_{12}}$
Te	0.00523(5)	0.00558(5)	0.00475(5)	-0.00023(3)	0.00148(3)	-0.00012(3)
Ba	0.00669(5)	0.00797(5)	0.00484(5)	0.00042(2)	0.00127(5)	0.00044(2)
O1	0.0062(5)	0.0154(6)	0.0121(6)	-0.0020(4)	0.00361(4)	-0.0003(4)
O2	0.0122(5)	0.0076(5)	0.0073(5)	0.0022(4)	0.00218(4)	0.0009(4)
O3	0.0102(5)	0.0088(5)	0.0069(5)	-0.0011(4)	0.0011(4)	-0.0004(4)
